# Determination of bioactive profile of *Polygala vulgaris L*. by GC-MS and molecular docking

**DOI:** 10.1186/s13065-025-01606-y

**Published:** 2025-08-14

**Authors:** Fatma Gönül Sezgin

**Affiliations:** Department of Biology, Faculty of Science, Ondokuz Mayıs University, Samsun, Türkiye

**Keywords:** *Polygala vulgaris*, GC-MS, Molecular docking, Oxidative stress, LIMP 2

## Abstract

Plants, being immobile, are constantly exposed to various biotic and abiotic stresses, leading to the generation of reactive oxygen species (ROS) which disrupt cellular homeostasis and cause oxidative damage. To combat this, plants have evolved various enzymatic and non-enzymatic antioxidant mechanisms. This study investigates the enzymatic activities of key antioxidants in *Polygala vulgaris L*., a species within the Polygalaceae known for its medicinal properties. The enzymatic activities of), catalase (CAT), superoxide dismutase (SOD), manganese superoxide dismutase (MnSOD) and phenol oxidase (PO) were measured. Additionally, the content of malondialdehyde (MDA), a marker for oxidative stress, was determined. The methanol extract of *P. vulgaris L*. was analyzed using GC-MS to identify bioactive compounds, and molecular docking studies were conducted to assess the interactions and binding energies of these compounds with the lysosomal integral membrane protein-2 (LIMP 2). LIMP 2 interaction of the extract suggested that it may trigger Parkison’s disease and at the same time protect the organism from enterovirus invasion. Results indicated significant antioxidant activity in *P. vulgaris L.*, with potential implications for its use in mitigating oxidative stress-related cellular damage. The study provides a comprehensive overview of the antioxidant defense mechanisms in *P. vulgaris L*. and underscores its potential for future pharmacological applications.

## Introduction

Since plants cannot change location throughout their lives, they are exposed to many biotic and abiotic stresses at their location. These environmental stress factors cause disruption of homeostasis and ion balance in the plant’s cells. Thus, osmotic stress is triggered, and reactive oxygen species (ROS) occur [1, 2]. Cell biochemistry is also the main source of ROS production. While chloroplast and mitochondria are ROS production centers, cell wall, cell membrane, endoplasmic reticulum and apoplast are other ROS production areas [3, 4]. Plants have developed various mechanisms to cope with these molecules that cause oxidative stress, such as antioxidant production and activation of stress response pathways [5]. Cells are equipped with a variety of enzymatic and non-enzymatic antioxidants that play a crucial role in scavenging excess ROS.

The genus *Polygala*, which is a key representative of the Polygalaceae, contains over 600 species globally. About 40 of these species are found in China. In Türkiye, there are 17 species of *Polygala*, three of which are endemic [6]. *P. vulgaris L.*, commonly distributed throughout Europe and Siberia, is a perennial herb characterized by a woody caudex. The species exhibits numerous ascending to erect stems. The lower leaves are narrowly obovate to spatulate, while the upper leaves are lanceolate to linear-lanceolate, and approximately the same length as the lower leaves. The inflorescences are exclusively terminal and lax. Bracts are typically shorter than the pedicels. The inner sepals, which are ovate to narrowly ovate and 6–7 mm in length, exceed the corolla and display blue, pink, or white coloration with anastomosing veins. The capsule is obcordate and narrower than the inner sepals. *P. vulgaris L*. commonly inhabits grasslands, meadows, and open woodlands, favoring well-drained soils. Its striking flowers and robust nature contribute to its use in traditional medicine and its appeal in naturalized wildflower meadows and gardens [7].

**Fig. 1:**
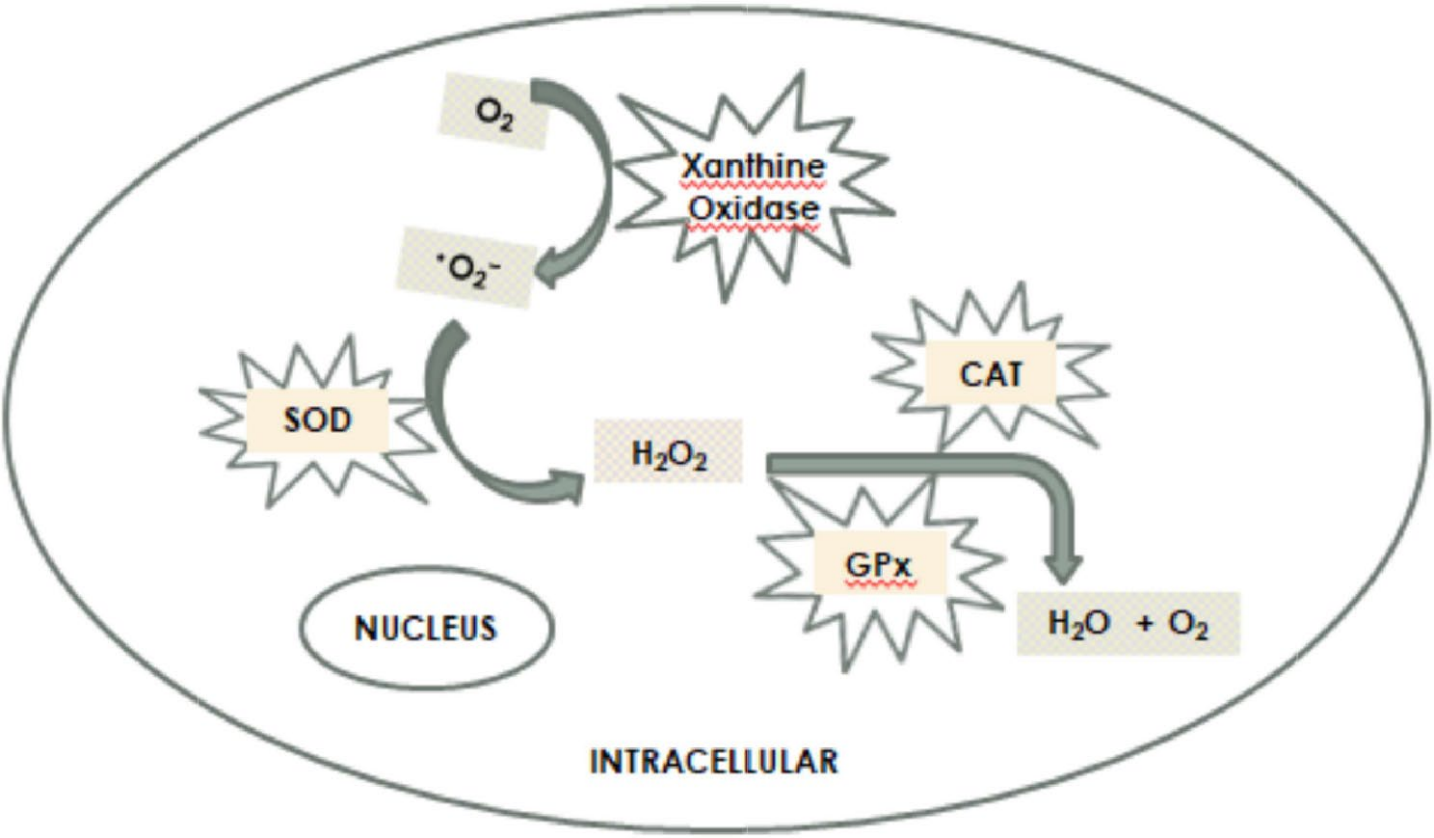
Formation of superoxide radical and the effects of intracellular antioxidant enzymes on superoxide radical [10]

Superoxide dismutase (SOD), one of the antioxidant enzymes in plants, oxidizes one superoxide radical to O2 molecule while simultaneously catalyzing the reduction of another superoxide radical to a less reactive molecule, hydrogen peroxide (H2O2). SOD is one of the main enzymatic systems that scavenge radicals created by stress in plants. SOD has Cu/Zn-SOD, Mn-SOD and Fe-SOD isoforms depending on the co-factors (Cu, Zn, Mn, Fe and Ni) it contains. Mn-SOD is in the mitochondria matrix and peroxisomes (Fig. 1) [8]. ^•^O_2_^−^ formed as a result of reactions in mitochondria is first transformed by Mn-SOD and GPX enzymes. However, a significant amount of H_2_O_2_ escapes from mitochondria and passes into the cytoplasm [9].

These escaped H_2_O_2_ molecules passing into the cytosol are detoxified by the CAT enzyme synthesized by peroxisomes.

CAT, a heme protein that converts hydrogen peroxide into water and oxygen, works in close synchrony with SOD to prevent the formation of reactive oxygen species. CAT, which converts H_2_O_2_ into water (H_2_O) and molecular oxygen (O_2_) is divided into three groups in plants according to their structure. The 1st group of CATs is found in photosynthetic tissues, the 2nd group in vascular tissues, and the 3rd group in seeds and young plants. Increased CAT activity is thought to be an adaptive feature focused on protecting tissue from metabolic damage by reducing harmful H_2_O_2_ levels [10].

**Fig. 2:**
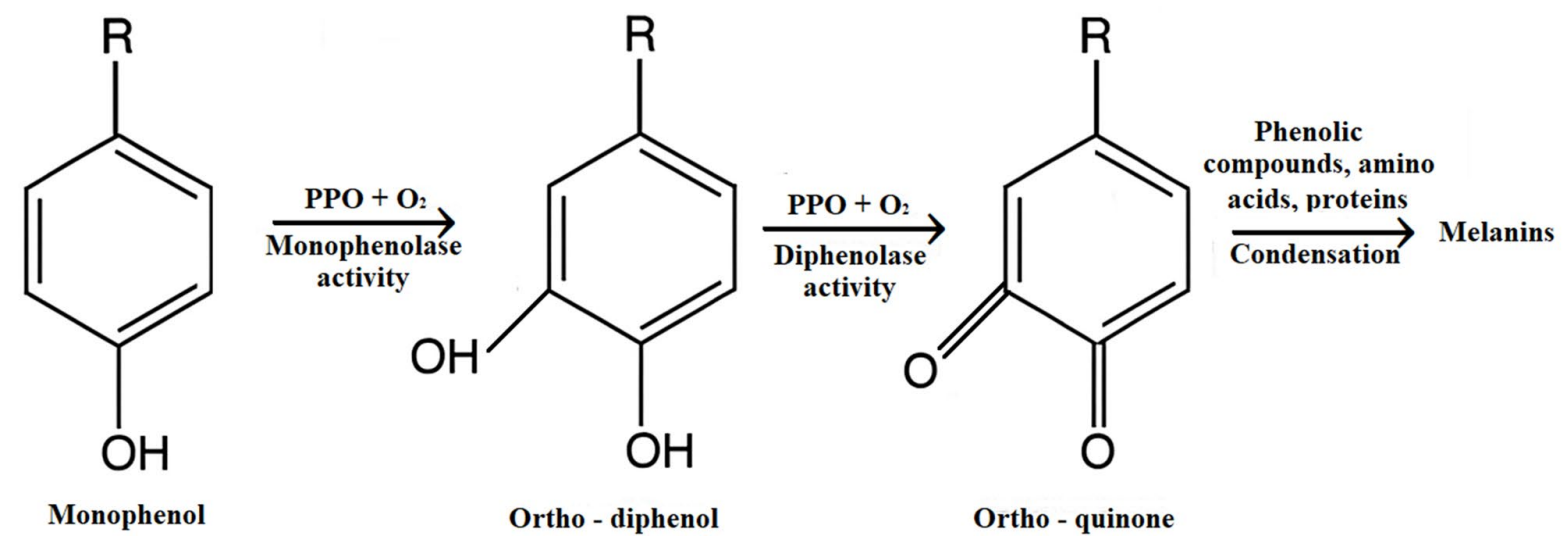
PPO catalyzes monophenol to o-quinone and then catalyzes pigment formation by polymerization of o-quinone

Phenol oxidase (PO) is found in higher plants, animals, and fungi. Due to the lack of PO studies in plants, the activation mechanism has not yet been elucidated. However, it is known that PO has a very important role in plant stress resistance and physiological metabolism (Fig. 2) [11]. PO has two important reactions: one is the reaction in which PO hydroxylates monophenol to o-diphenol, and the other is It _is_ the reaction in which o-diphenol is oxidized to o-quinone. o-quinone is further polymerized and condensed with amino acids and proteins to produce brown substances [12].

It has been determined that PO, in addition to enzymatic browning, is one of the enzymes that has a very important role in plant physiology [13] and plant immunity in terms of resistance of plants against microorganisms and herbivorous insects [11].

Malondialdehyde (MDA) content is a widely used parameter that expresses lipid peroxidation and redox signaling that occur in case of oxidative stress in plant physiology [14]. MDA is a damage indicator product that occurs as a result of the oxidation of polyunsaturated fatty acids in cells and their structure deterioration [15].

LIMP 2 transports the enzyme glucocerebrosidase (GCase), which breaks down glucocerebrosides and is responsible for maintaining lysosomal function, to the lysosome. Mutations in GCase cause Gaucher disease and GCase -associated Parkinson’s disease. GCase cannot be transported to the lysosome, resulting in lysosomal dysfunction and a-synuclein accumulation. a-synuclein accumulation is the fundamental pathological feature of Parkinson’s disease. LIMP 2, which also plays a role in intracellular lipid homeostasis and autophagy, may serve as a receptor or accessory protein for the entry of some enteroviruses into the cell.

The binding of the virus to this protein facilitates its entry into the cell via the endosomal pathway [16–19].

While the effects of *P. altomontana*, *P. caudata*,* P. flavescens*,* P. glomerata*,* P. japonica*, *P. molluginifolia*,* P. sibirica*,* P. tenuifolia* species have been demonstrated in the Chinese medicine system for use in traditional medicine in the last few years [6, 7], the fact that such properties of *P. vulgaris L.* are unknown has been the subject of this study. Therefore, the aim of this study was to identify phytochemical compounds found in methanol extracts of *P. vulgaris L.* by GC-MS method, to determine potential LIMP2 interaction by molecular docking method and to determine the properties of potential antioxidant bioactive compounds. This study will provide valuable information about the bioactivity of compounds obtained from *P. vulgaris L*. and support the discovery of new therapeutic agents.

## Experimental

### Plant materials

Plant materials of *P. vulgaris L*. were collected between June and August from various grassland locations in Samsun province. These locations included the Büyük Gölet at Ondokuz Mayıs University Campus, and the districts of Kavak, Havza, and Çarşamba. Complete specimens, encompassing roots, stems, leaves, flowers, fruits, and seeds, were gathered. Dr. Alper Durmaz identified the samples using the Flora of Türkiye, and the current taxonomic status was verified through the Plants of the World Online (POWO) database. The species is recorded in the Ondokuz Mayıs University Herbarium with the accession number OMUB-2435. After collection, the plants were cleaned to remove soil and foreign materials. They were then placed in a drying oven in the Plant Material and Soil Preparation Laboratory.

#### Plant extraction

The aboveground parts of *P. vulgaris L*. dried in an oven at 40 °C were pulverized using a blender. The maceration method suggested by Aytar (2023) [20] was used for extraction of the sample. The dried and pulverized aboveground parts were extracted by keeping them in methanol in a dark environment for two days. Then, they were filtered with filter paper. Then, the solvent was evaporated under reduced pressure using a rotary evaporator at 40° C. The solid extracts obtained by evaporation were stored at + 4° C.

### GC-MS analysis

Samples centrifuged at 3500 rpm for 10 min were subjected to GC and MS analyses with a column flow of 1 ml/min [20]. Analyses using the NIST Standard Reference Database were performed in accordance with the protocol.

### Molecular docking studies

Minimum energy forms of molecules drawn in Chem-Draw Ultra 18.0 program were obtained in Chem 3D 18.0 program. obtained data were saved in Mol2 format. Protein Data Bank (PDB) was used to save enzymes. The structure of LIMP 2 (space group C2221) was selected as PDB-ID 4Q4B (2.82 Å) and deposited in PDB format. Molecule-enzyme interactions were determined using AutoDock Vina 1.5.7 software and binding energies (kcal/mol) were determined [21]. 2D and 3D images were displayed with BIOVIA Discovery Studio Visualizer software [22].

### Enzyme activity assays

#### Superoxide dismutase (SOD) activity

SOD activity was determined by modifying the spectrophotometric methods of [23, 24]. This method is based on the inhibition of cytochrome C reduction by superoxide radicals released by the xanthine/xanthine oxidase reaction. This inhibition is followed spectrophotometrically at 550 nm. For the experiment, 0.76 mg (5 µl) of xanthine was dissolved in 10 ml of 0.001 N NaOH and 24.8 mg (2 µmol) of cytochrome C was dissolved in 100 ml of 50 mM phosphate buffer (pH 7.8) containing 0.1 M EDTA. This solution is stable for 3 days at + 4 °C. 20 µl of extract was added to this 1 ml mixture in the spectrophotometer cuvette. Then, the reaction was started by adding 0.2 U/ml xanthine oxidase. The blank sample was prepared in the same way as the sample, but instead of the extract, 20 µl of phosphate buffer was added to the cuvette. The reaction tube without extract was used as the control reaction system. All measurements were repeated 3 times (*n* = 3). The change in absorbance was monitored for two minutes. One unit of SOD activity is defined as the amount of enzyme that inhibits the reduction of cytochrome C by 50%. The amount of enzyme that inhibits the reduction of cytochrome C by 50% is defined as one unit of SOD activity.

#### Manganese superoxide dismutase (MnSOD) activity

MnSOD activity was determined using the same method as SOD activity, with the addition of 10 µl of 10 mM NaCN to solution A, followed by a 20-minute incubation. The activity was then determined by adding 0.2 U/ml xanthine oxidase [23, 24].

#### Catalase (CAT) activity

Catalase activity was determined using the method of [25]. This method is based on the spectrophotometric measurement of the decrease in absorbance at 240 nm due to the breakdown of H2O2 by catalase. For the assay, a phosphate buffer (pH 7, 1/15 M) was prepared by mixing Na_2_HPO4.H_2_O and KH_2_PO4. To 100 ml of this buffer, 160 µl of H2O2 was added. The reaction was initiated by adding 20 µl of the extract, and the change in absorbance was monitored for 2 min. The enzyme unit was calculated according to the change in absorbance; the amount of enzyme that catalyzes the conversion of 1 µmol H2O2 per minute was defined as one unit of catalase activity.

#### Phenole oxidase (PO) activity

PO activity was determined using the method of [26]. The extract was added to a phosphate buffer containing 20 mM L-DOPA, and the change in absorbance was measured at 492 nm. The activity PO in the sample was calculated in PO units, where one unit is defined as the amount of enzyme that increases the absorbance by 0.001 per minute.

#### Malondialdehyde (MDA) activity

Lipid peroxidation activity was analyzed according to the methods of [27]. For this analysis, 10% trichloroacetic acid (TCA) and 0.675% thiobarbituric acid (TBA) solutions were used. Malondialdehyde (MDA), a product of lipid peroxidation, forms a pink complex with TBA when incubated at 90 °C. The absorabance of this complex was measured at 532 nm. The MDA values were calculated in nmol/ml using the extinction coefficient of the MDA-TBA complex at 532 nm (*n* = 1.56 × 10^5 cm^-1 M^-1).

### Statistical analysis

The statistical analysis of the data was conducted using SPSS 22. Descriptive Statistical Analysis and Student’s t-test application was used to analyze the confidence interval.

## Results

### Enzymatic activity and amount of MDA

The enzymatic activity and corresponding standard deviations for various enzymes were measured. SOD exhibited an activity of 41.52 IU/ml with a standard deviation of ± 1.2755. MnSOD displayed an activity of 6.35 IU/ml, with a standard deviation of ± 0.2157. MnSOD activity located in the mitochondria matrix of *P. vulgaris L* accounts for approximately 15% of the total SOD activity.

CAT activity was recorded at 242.35 IU/ml, accompanied by a standard deviation of ± 3.9326. PO showed an activity level of 12.695 IU/ml and a standard deviation of ± 1.0841. Additionally, the MDA concentration was determined to be 12.695 × 10^−^⁷ M, with a standard deviation of ± 1.841 × 10^−^⁷ M (Table 1). These findings provide a detailed overview of the enzymatic activities and their variability in the given sample.

**Table 1:** Descriptive statistical analysis of SOD, mnsod, CAT, PO activities and MDA amount

	Minimum	Maximum	Median	Standard deviation	Variance
SOD	40,2445 IU/ml	42,7955 IU/ml	41.52 IU/ml	1,2755 IU/ml	1,6279
MnSOD	6,134 IU/ml	6,566 IU/ml	6,35 IU/ml	0,2157 IU/ml	0,0465
CAT	238,42 IU/ml	246,28 IU/ml	242,35 IU/ml	3,9326 IU/ml	15,46
PO	11,881 IU/ml	14,049 IU/ml	12,965 IU/ml	1,0841 IU/ml	1,1752
MDA	1,0854 × 10⁻⁶ M	1,4536 × 10⁻⁶ M	1,2695 × 10⁻⁶ M	1,8411 × 10⁻⁷ M	3,3906 × 10⁻¹⁴

These measurements show that the activities of SOD, MnSOD, CAT and PO enzymes and the amount of MDA are consistent and biologically significant under experimental conditions. The wide 95% confidence intervals for all enzyme activities and MDA values ​​may indicate the need for more samples to increase statistical robustness.

### GC-MS analysis results

The concentration (% area), retention time (RT) and chemical structure of 7 bioactive phytochemical compounds obtained from the methanol extract of *P. vulgaris L.* are presented in Table 2. The major components identified in the aerial parts of *P. vulgaris L*. were 3’,5’-Dimethoxyacetophenone (46.15%), D-Glucitol, 1,5-Anhydro- (41.16%), N-Isobutyl-(2E,4Z,8Z,10E)-dodecatetraenamide (5.61%), 2,2-dimethoxybutane (2.46%), beta-D-Glucopyranose, 1,2,3,4,6-pentakis-O-(trimethylsilyl)- (1.16%), Methyl butyrate (0.58%) and Malonic acid (0.24%).

**Table 2:**
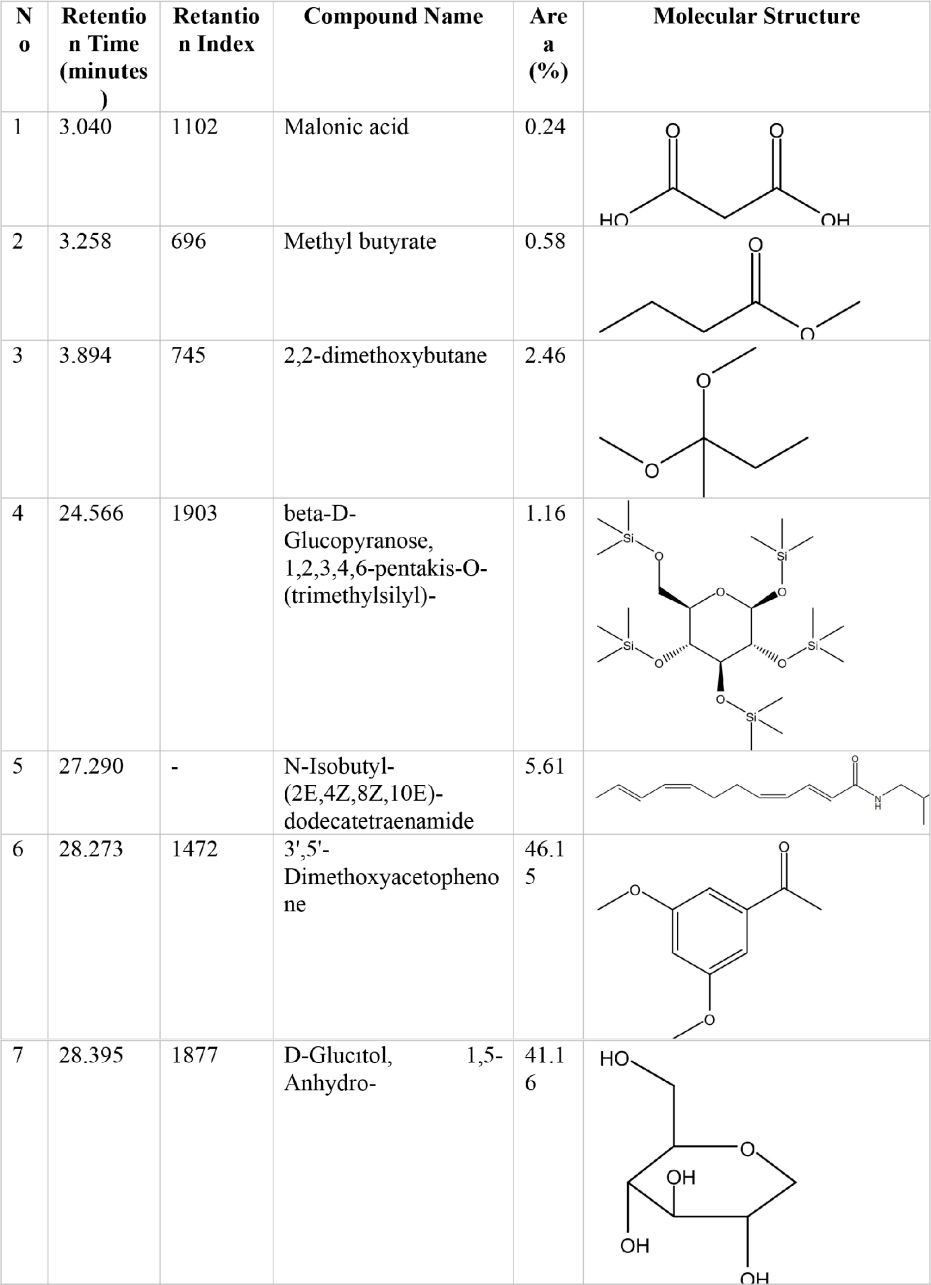
GC-MS analysis results of *P. vulgaris L*

### Molecular docking study

**Fig. 3:**
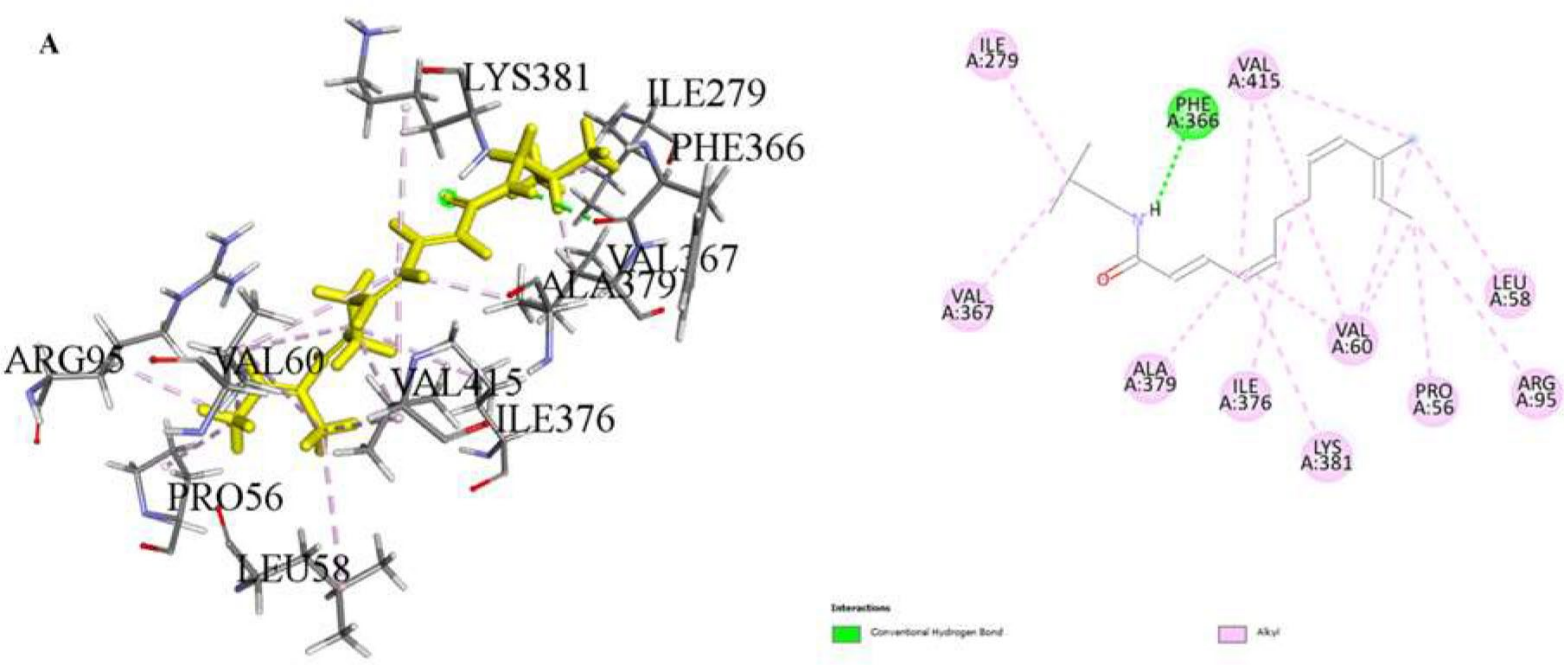
Molecular docking process of **A**) N-Isobutyl-(2E,4Z,8Z,10E)-dodecatetraenamide with LIMP-2 (space group C2221)

**Fig. 4:**
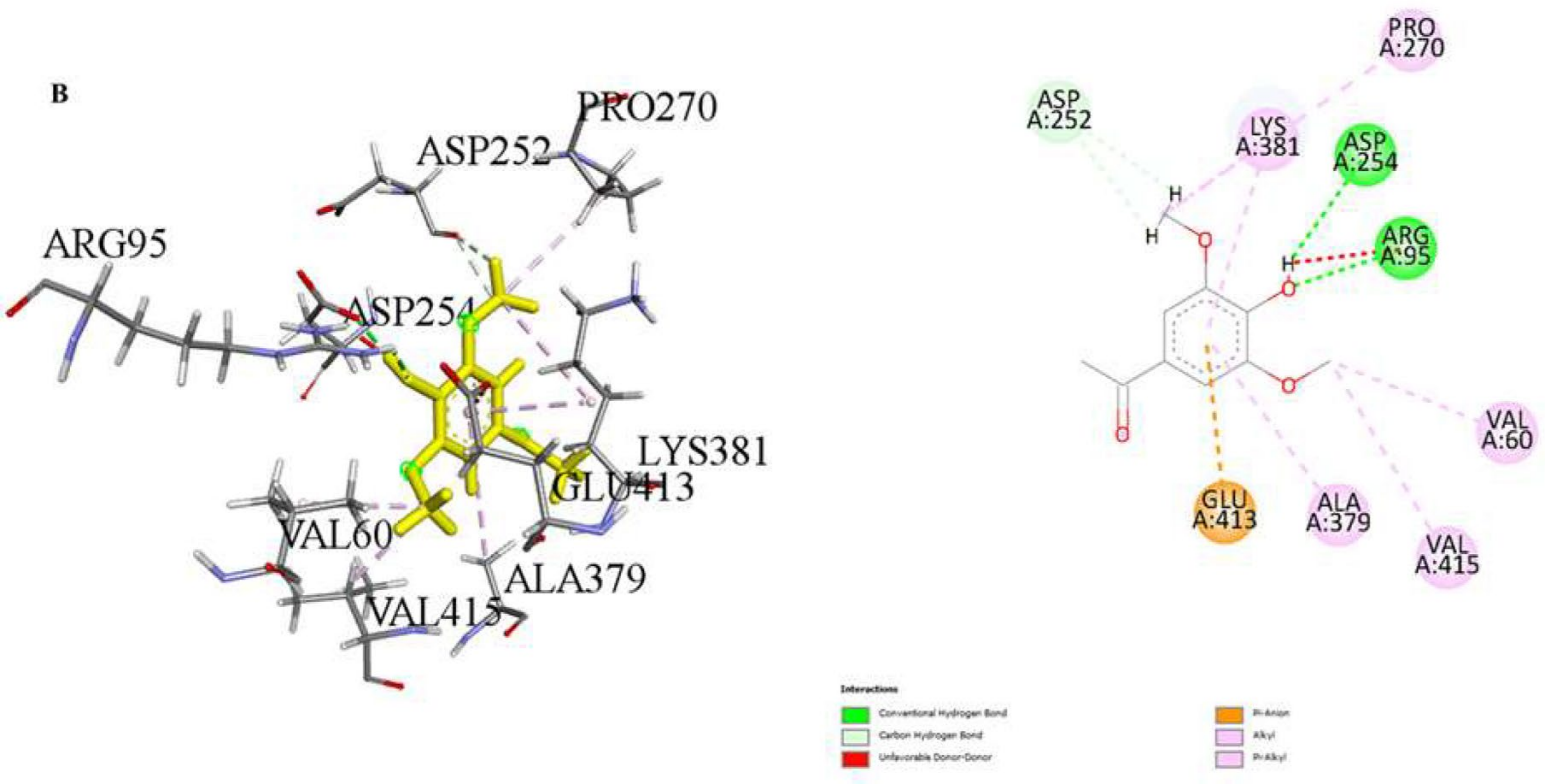
Molecular docking process of **B**) 3’ 5’-dimethoxy-4’-hydroxyacetophenone with LIMP 2 (space group C2221)

**Fig. 5:**
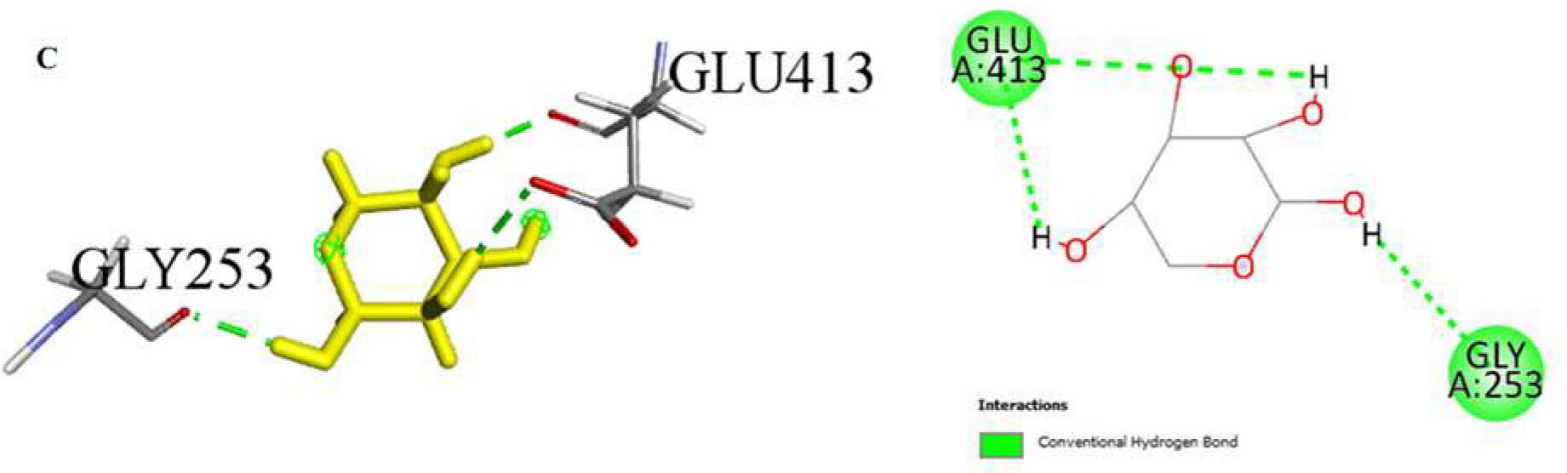
Molecular docking process of **C**) 1,5-Anhydro-D-glucitol with LIMP 2 (space group C222bi

Our molecular docking study evaluated the binding energies of specific amino acids along with the potential binding sites of various organic compounds in our extract. The findings provide insight into potential biological interactions and the binding properties of these interactions. (Table 3; Figs. 3, 4 and 5). In our study, simulations of 2D and 3D interactions between molecules and the LIMP 2 enzyme (space group C2221) were conducted in silico, as illustrated in the Figures. Additionally, the interactions between the compounds and the enzyme in the optimal binding positions are presented in the Table 3. The compounds 1,5-Anhydro-D-glucitol (-5.4 kcal/mol), 3’,5’-Dimethoxy-4’-hydroxyacetophenone (-5.6 kcal/mol), and N-Isobutyl-(2E,4Z,8Z,10E)-dodecatetraenamide (-4.4 kcal/mol) exhibited the best interaction scores with LIMP 2.

In this study, the interactions, and binding energies of three different compounds with a protein were investigated: 1,5-Anhydro-D-glucitol, 3’,5’-Dimethoxy-4’-hydroxyacetophenone, and N-Isobutyl-(2E,4Z,8Z,10E)-dodecatetraenamide. These compounds form various types of bonds with amino acids, and the distances of these bonds were determined. The binding energy for 1,5-Anhydro-D-glucitol was calculated to be -5.4 kcal/mol. This compound forms three conventional hydrogen bonds with GLY253 and GLU413, with distances of 2.00, 2.29, and 1.62 Å, respectively. For 3’,5’-Dimethoxy-4’-hydroxyacetophenone, the binding energy is -5.6 kcal/mol. It forms two conventional hydrogen bonds with ARG95 and ASP254, and two carbon hydrogen bonds with ASP252, with bond distances of 2.05, 2.13, 2.71, and 2.90 Å, respectively. Additionally, there is a Pi-Anion interaction with GLU413 and various alkyl interactions with VAL60, VAL415, and PRO270. N-Isobutyl-(2E,4Z,8Z,10E)-dodecatetraenamide, with a binding energy of -4.4 kcal/mol, forms one conventional hydrogen bond with PHE366 (distance of 3.09 Å) and several alkyl interactions with amino acids such as VAL60, ALA379, LYS381, VAL415, ILE376, ILE279, VAL367, LEU58, PRO56, and ARG95.

A comprehensive analysis of these compounds’ interactions with the protein provides valuable insights into their potential biological activities and interaction mechanisms. The binding energies and types of interactions for each compound offer detailed information about their affinities for the binding sites on the protein surface. These data form a critical foundation for drug design and understanding protein-ligand interactions.

**Table 3:** Report on the interaction of phytochemicals with LIMP 2

Component	Binding Energy (kcal/mol)	Amino acid	Interacting	Distance	Interaction Type
1,5-Anhydro-D-glucitol	-5.4	H1 - A: GLY253:O	Conventional Hydrogen Bond	2.00	Hydrogen bond
		H2 - A: GLU413:OE1	Conventional Hydrogen Bond	2.29	Hydrogen bond
		H4 - A: GLU413:O	Conventional Hydrogen Bond	1.62	Hydrogen bond
3’ 5’-dimethoxy-4’-hydroxyacetophenone	-5.6	A: ARG95:HH22	Conventional Hydrogen Bond	2.05	Hydrogen bond
		H4- A: ASP254:OD1	Conventional Hydrogen Bond	2.13	Hydrogen bond
		H10- A: ASP252:O	Carbon Hydrogen Bond	2.71	Hydrogen bond
		H12- A: ASP252:O	Carbon Hydrogen Bond	2.90	Hydrogen bond
		A: GLU413:OE1	Pi-Anion	4.00	π–Anion interaction
		C6- A: VAL60	Alkyl	4.34	Alkyl
		C6- A: VAL415	Alkyl	4.06	Alkyl
		C10- A: PRO270	Alkyl	5.19	Alkyl
N-Isobutyl-(2E,4Z,8Z,10E)-dodecatetraenamide	-4.4	H1- A: PHE366:O	Conventional Hydrogen Bond	3.09	Hydrogen bond
		A: VAL60	Alkyl	4.75	Alkyl
		A: VAL60	Alkyl	5.28	Alkyl
		A: ALA379	Alkyl	3.83	Alkyl
		A: LYS381	Alkyl	5.18	Alkyl
		A: VAL415	Alkyl	4.26	Alkyl
		A: VAL415	Alkyl	4.43	Alkyl
		A: ILE376	Alkyl	4.93	Alkyl
		C13- A: ILE279	Alkyl	4.60	Alkyl
		C13- A: VAL367	Alkyl	3.51	Alkyl
		C16- A: LEU58	Alkyl	5.17	Alkyl
		C16- A: VAL60	Alkyl	5.31	Alkyl
		C16- A: VAL415	Alkyl	4.30	Alkyl
		C17- A: PRO56	Alkyl	4.33	Alkyl
		C17- A: VAL60	Alkyl	4.05	Alkyl
		C17- A: ARG95	Alkyl	4.51	Alkyl

## Discussion

This study represents one of the first comprehensive investigations on the phytochemical properties and antioxidant defense system of *P.vulgaris L*. In this study, the phytochemical components of the methanol extract of *P. vulgaris L*. were analyzed by GC-MS method, in silico molecular docking study was performed and SOD, MnSOD, CAT, PO antioxidant system enzyme activities and metabolic MDA amount were determined.

It was determined that especially 3’,5’-Dimethoxyacetophenone (%46.15), 1,5-Anhydro-D-glucitol (%41.16) and N-Isobutyl-(2E,4Z,8Z,10E)-dodecatetraenamide (%5.61) found in high amounts in the plant extract showed significant interaction with LIMP 2 protein.

In molecular docking studies, predicted binding free energies (ΔG) are useful as initial filters but require context-specific thresholds to extract potential biological relevance. According to the AutoDock Vina rule, ligands that exhibit affinities more negative than approximately − 7.0 kcal/mol are generally considered “lead compounds.” Values ​​between − 5 and − 7 kcal/mol are considered “moderate.” Values ​​weaker than − 5 kcal/mol are generally not prioritized. However, this threshold can vary depending on the target protein, the size and flexibility of the ligand, and the scoring function used [28].

LIMP 2 directly increases the activity of GCase, a lysosomal enzyme involved in the degradation of the sphingolipid glucosylceramide (GluCer). Deficiencies in the activity of the GCase enzyme have been associated with Parkinson’s disease (PD) [16, 18, 19]. Our results are in line with the hypothesis that preserving LIMP 2 activity in Parkinson’s disease may have a neuroprotective effect.

However, another study has also linked the LIMP 2 protein to the formation of foam cells [29]. Another study has shown that LIMP 2 is the receptor of enterovirus 71 [30].

Ligands that exhibit favorable binding energies in docking studies could potentially enhance the LIMP 2–GCase interaction, which could increase lysosomal targeting of GCase or reduce α-synuclein accumulation via transmembrane modulation.

Molecular docking analyses revealed that 3′,5′-dimethoxyacetophenone and 1,5-anhydro-D-glucitol bind to LIMP 2 with binding energies of -5.6 kcal/mol and − 5.4 kcal/mol, respectively. These findings suggest that these compounds have potential interaction affinity for LIMP 2. However, small molecules with moderate affinity that disrupt the GCase–LIMP 2 interaction may exacerbate neuronal lysosomal dysfunction and intensify Parkinsonian symptoms. Therefore, detailed analysis of the interaction is critical.

In addition, it was determined that the plant extract had high levels of SOD (41.52 IU/ml), MnSOD (6.35 IU/ml), CAT (242.35 IU/ml) and PO (12.965 IU/ml) enzyme activities. These enzymes are the main antioxidant systems involved in the elimination of reactive oxygen species (ROS). Especially, high CAT activity indicates that intracellular H₂O₂ levels were effectively detoxified and cellular oxidative stress was reduced [31]. At the same time, the low level of MDA (12.965 × 10⁻⁷ M) in the extract revealed that lipid peroxidation was limited and at the same time suggested the activity of antioxidant systems.

LIMP 2 dysfunction may cause lysosomal dysfunction and consequently oxidative stress accumulation as it reduces GCase activity. Cleaning of reactive oxygen species may slow down the progression of Parkinson’s by preserving the LIMP 2-GCase interaction. The antioxidant effect of the plant extract will also support antiviral defense and suppress the entry of viruses that bind to LIMP 2 into the cell. Thus, in addition to its antioxidant effect; it also shows antiviral effect for enterovirus and similar viruses. It has been reported that 3’,5’-Dimethoxyacetophenone, which is found in high amounts in the plant extract, inhibits aldose reductase, causes collagenase, and has anticancer effects on human leukemic cells [32]. In one study, acetophenones and their derivatives have been reported to have many biological activities such as antioxidant, analgesic, cardioprotective, neuroprotective and antidiabetic [33]. In other studies, it has been reported that acetophenones and their derivatives have a wide area of ​​use in cosmetics [34] because they have good antiseptic properties, and in the production of sunscreens because they provide good UV light protection [35]. These compounds, which are considered very valuable for medicinal chemistry, also have very wide biological properties.

1,5-Anhydro-D-glucitol is a form of 1-deoxy glucose discovered in Polygala senega in 1888 [36]. This compound reduces oxidative stress-related damage and glucose uptake into the cell [37]. At the same time, its sugar alcohol structure exhibits antioxidant properties by binding free radicals [38]. N-Isobutyl-(2E,4Z,8Z,10E)-dodecatetraenamide is an alkyamide and, like other alkylamides, it has been reported to have anticancer, neuroprotective, analgesic, antioxidant, anti-inflammatory, antimutagenic, antimicrobial, insecticidal [39]. and antiparasitic effects in leishmaniasis and trypanosomiasis [40]. According to the results obtained, the extract of *P. vulgaris L.* contains compounds with significant antioxidant properties.

Although numerous pharmacological studies have been conducted on other species in the Polygala genus, research on P. vulgaris has been limited [7]. These properties of *P. vulgaris L*. obtained by GC-MS, in silico molecular docking studies and antioxidant enzyme activity analyses support the biochemical basis of its use in traditional medicine and suggest that the plant can be evaluated as a potential therapeutic agent in LIMP 2 related diseases such as Parkinson’s. However, it is important to confirm these effects observed at the molecular level in in vivo models and clarify the direction of action.

## Conclusion

This study demonstrates the potential of *P. vulgaris L*. to interact with LIMP 2 protein, indicating that it is an important candidate for alternative treatment studies in LIMP 2 related diseases such as Parkinson’s disease. The components identified by GC-MS analyses exhibited significant binding with LIMP 2 and showed high enzyme activities supporting antioxidant defense systems. These results indicate that *P. vulgaris L*. can be evaluated as an antioxidant and possible neuroprotective agent in protection against enterovirus and in oxidative stress-related diseases.

This study revealed the binding modes, binding affinity and cellular oxidative stress metabolism of *P. vulgaris L.* before its possible use in medical applications. Therefore, it is expected to shed light on the effectiveness of *P. vulgaris L.* in clinical studies or applications and to be a source for further studies comparing its antioxidant behavior. In this direction, it is important to confirm with in vivo studies and clarify the direction of effect.

## Data Availability

The datasets used and/or analysed during the current study are available from the corresponding author on reasonable request.
